# Supplementary material for: Japonamides A and B, Two New Cyclohexadepsipeptides from the Marine-Sponge-Derived Fungus Aspergillus japonicus and Their Synergistic Antifungal Activities
Source: J Fungi (Basel). 2022 Oct 9;8(10):1058. doi: 10.3390/jof8101058 (PMC9605600; doi:10.3390/jof8101058)
Supplement: Supplementary file 1 [file jof-08-01058-s001.zip › jof-1921224-suppl.pdf]

Supporting Information

Japonamides A and B, Two New Cyclohexadepsipeptides from the Marine-Sponge-Derived Fungus *Aspergillus japonicus* and Their Synergistic Antifungal Activities

Haifeng Wang [1,†], Rui Zhang [1,†], Ben Ma [1], Wenzhao Wang [2], Chong Yu [1], Junjie Han [2], Lingjuan Zhu [1], Xue Zhang [1], Huanqin Dai [2], Hongwei Liu [1,2,*] and Baosong Chen [2,*]

1 School of Traditional Chinese Materia Medica, Shenyang Pharmaceutical University, Shenyang 110016, China
2 State Key Laboratory of Mycology, Institute of Microbiology, Chinese Academy of Sciences, Beijing 100190, China
* Correspondence: liuhw@im.ac.cn (H.L.); chenbs@im.ac.cn (B.C.)
† These authors contributed equally to this work.

Table of contents

Figure S1 Advanced Marfey's analysis of compound **1**. 3
Figure S2 Advanced Marfey's analysis of compound **2**. 4
Figure S3 ^{1}H NMR spectrum of compound **1** in DMSO-d_6 5
Figure S4 ^{13}C NMR spectrum of compound **1** in DMSO-d_6 6
Figure S5 HSQC spectrum of compound **1** in DMSO-d_6 7
Figure S6 ^{1}H-^{1}H COSY spectrum of compound **1** in DMSO-d_6... 8
Figure S7 HMBC spectrum of compound **1** in DMSO-d_6 9
Figure S8 ROESY spectrum of compound **1** in DMSO-d_6... 10
Figure S9 ^{1}H NMR spectrum of compound **2** in DMSO-d_6 11
Figure S10 ^{13}C NMR spectrum of compound **2** in DMSO-d_6 12
Figure S11 HSQC spectrum of compound **2** in DMSO-d_6 13
Figure S12 ^{1}H-^{1}H COSY spectrum of compound **2** in DMSO-d_6.. 14
Figure S13 HMBC spectrum of compound **2** in DMSO-d_6 15
Figure S14 ROESY spectrum of compound **2** in DMSO-d_6.. 16

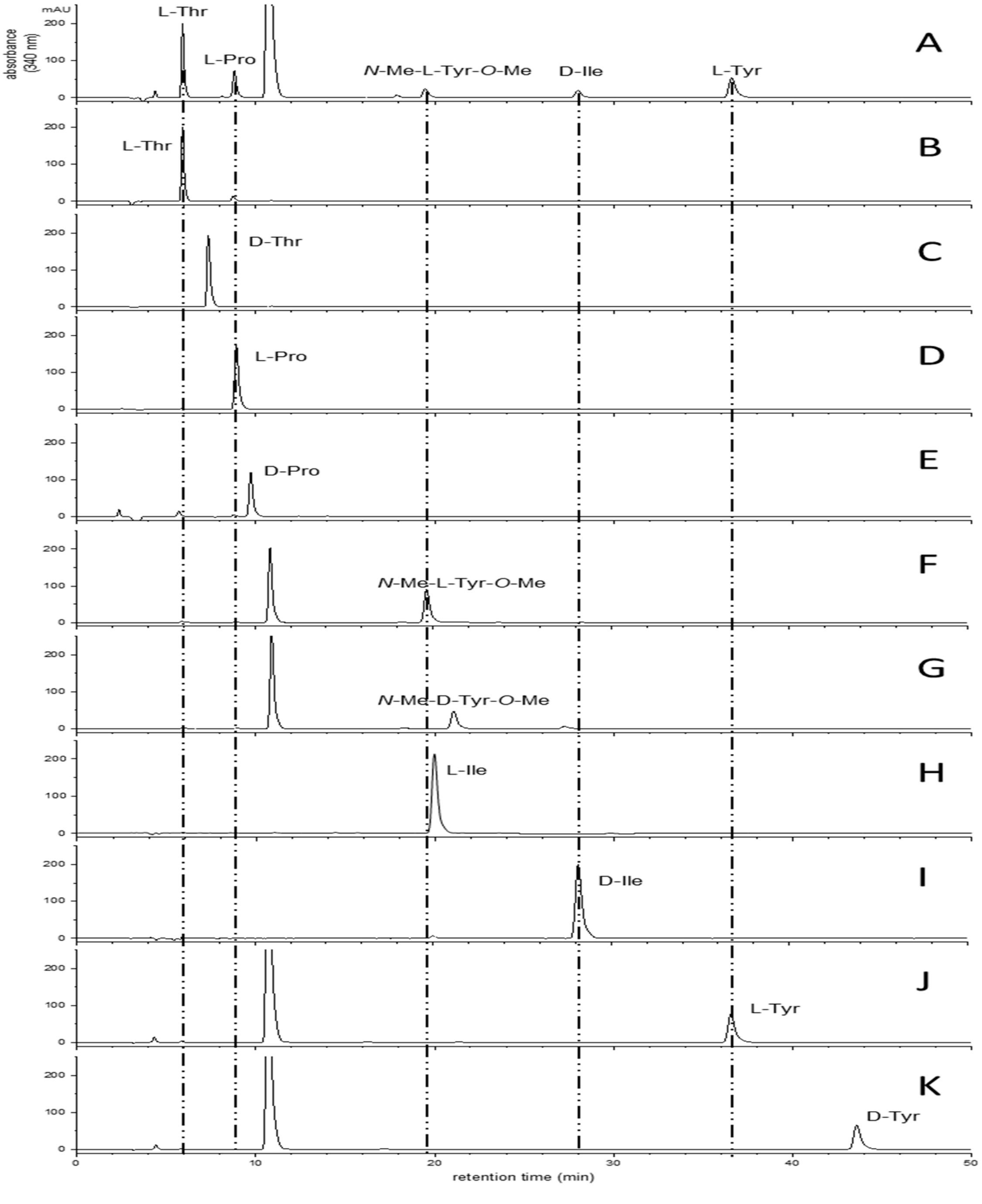

Figure S1 Advanced Marfey's analysis of compound **1**.

The FDAA derivatives of standards compouds **B-K** were L-Thr (5.9 min), D-Thr (6.9 min), L-Pro (8.8 min), D-Pro (9.6 min), *N*-Me-O-Me-L-Tyr (19.3 min), *N*-Me-*O*-Me-D-Tyr (21.2 min), L-Ile (19.9 min), D-Ile (28.1 min), L-Tyr (36.3 min) and D-Tyr (43.7 min), respectively. The derivatives of the acid hydrolysate and the standard amino acids were subjected to RP HPLC analysis (Kromasil C18 column; 5 µm, 4.6 × 250mm; 1.0 mL/min; UV detection t 340 nm) with a linear gradient of acetonitrile (30%-50%) in water (TFA, 0.01%) over 50 min.

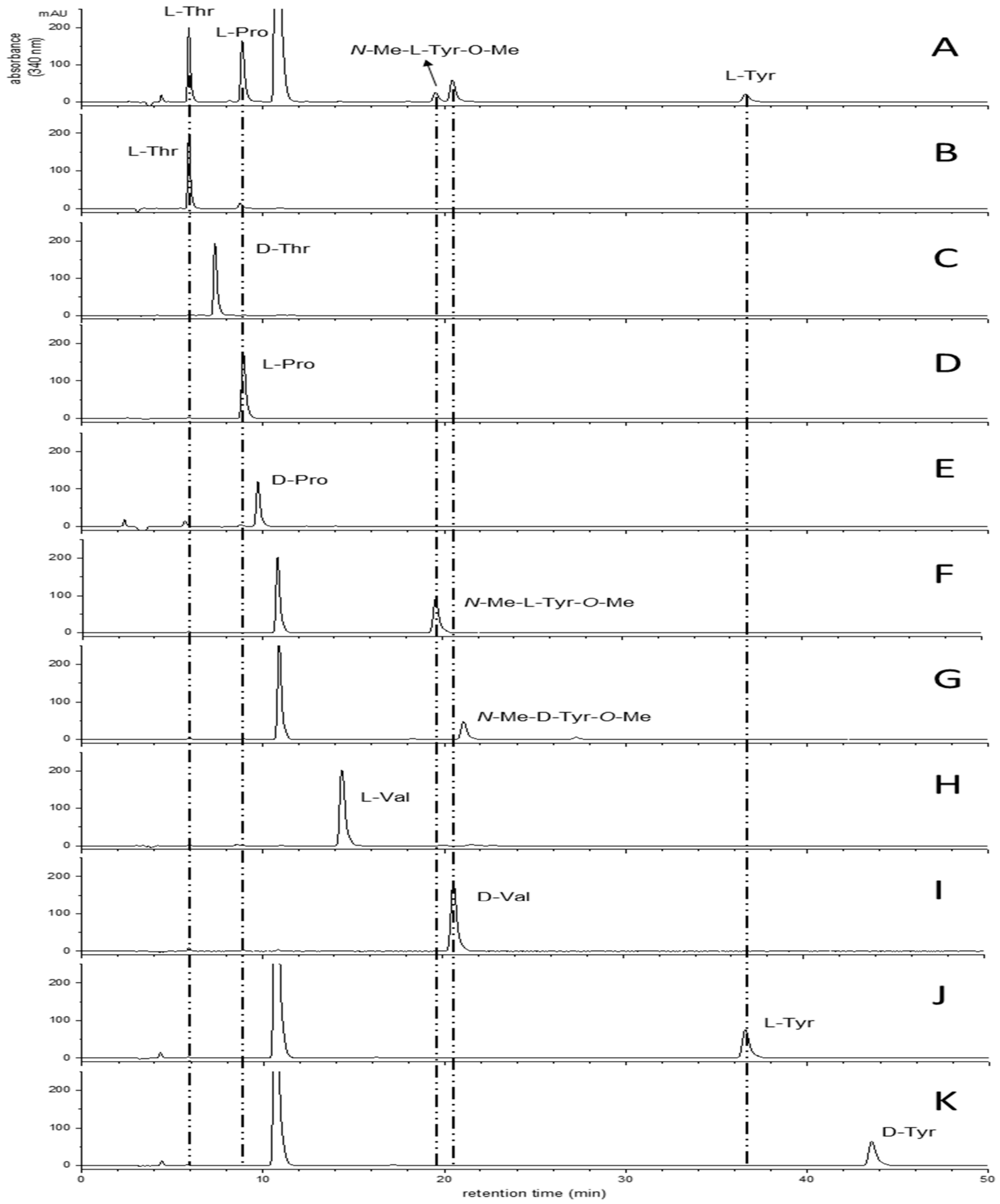

Figure S2 Advanced Marfey's analysis of compound **2**.

The FDAA derivatives of standards compouds **B-K** were L-Thr (5.9 min), D-Thr (6.9 min), L-Pro (8.8 min), D-Pro (9.6 min), *N*-Me-O-Me-L-Tyr (19.3 min), *N*-Me-*O*-Me-D-Tyr (21.2 min), L-Val (14.2 min), D-Val (20.5 min), L-Tyr (36.3 min) and D-Tyr (43.7 min), respectively. The derivatives of the acid hydrolysate and the standard amino acids were subjected to RP HPLC analysis (Kromasil C18 column; 5 µm, 4.6 × 250mm; 1.0 mL/min; UV detection t 340 nm) with a linear gradient of acetonitrile (30%-50%) in water (TFA, 0.01%) over 50 min.

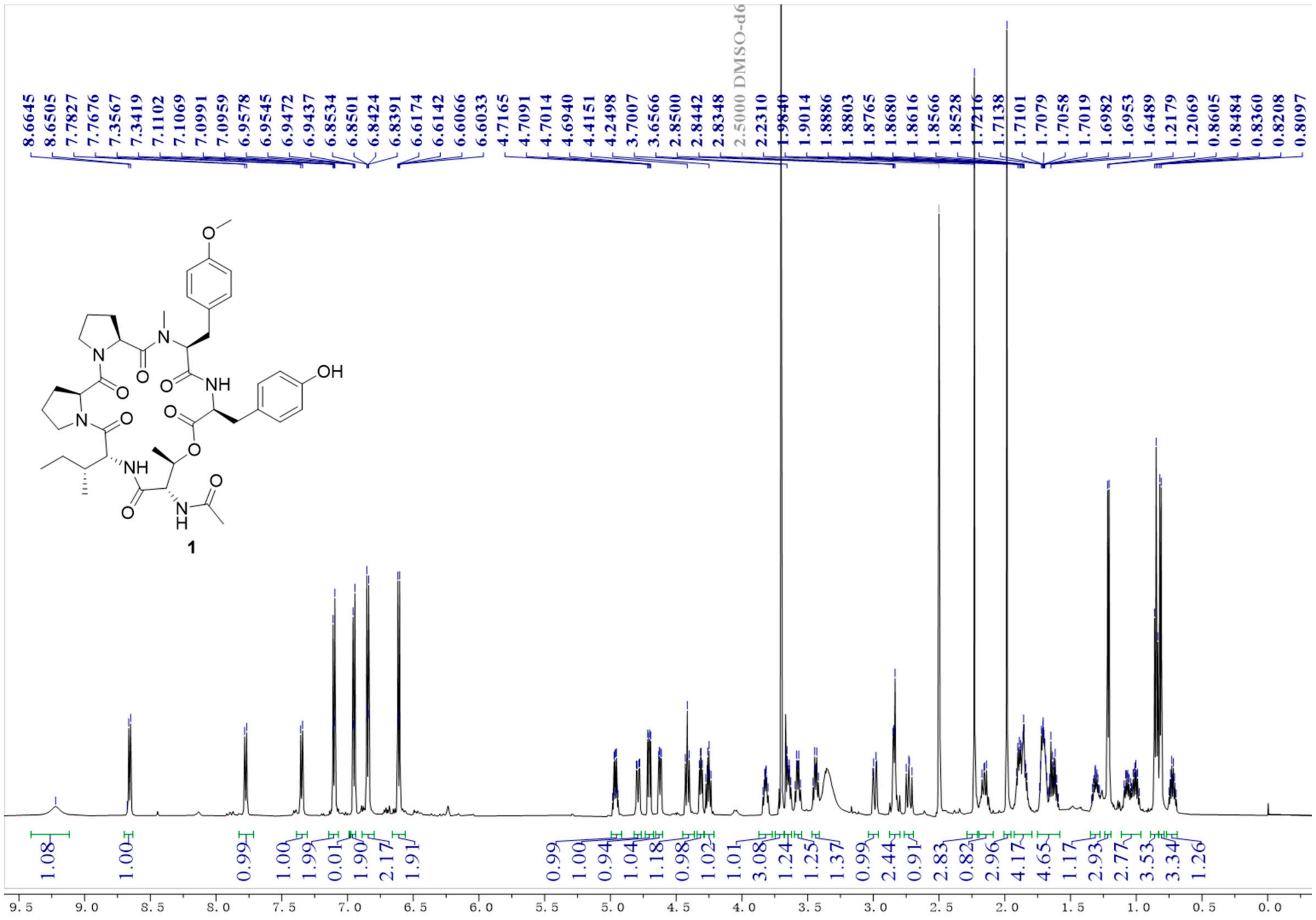

Figure S3 ^{1}H NMR spectrum of compound **1** in DMSO-d_6

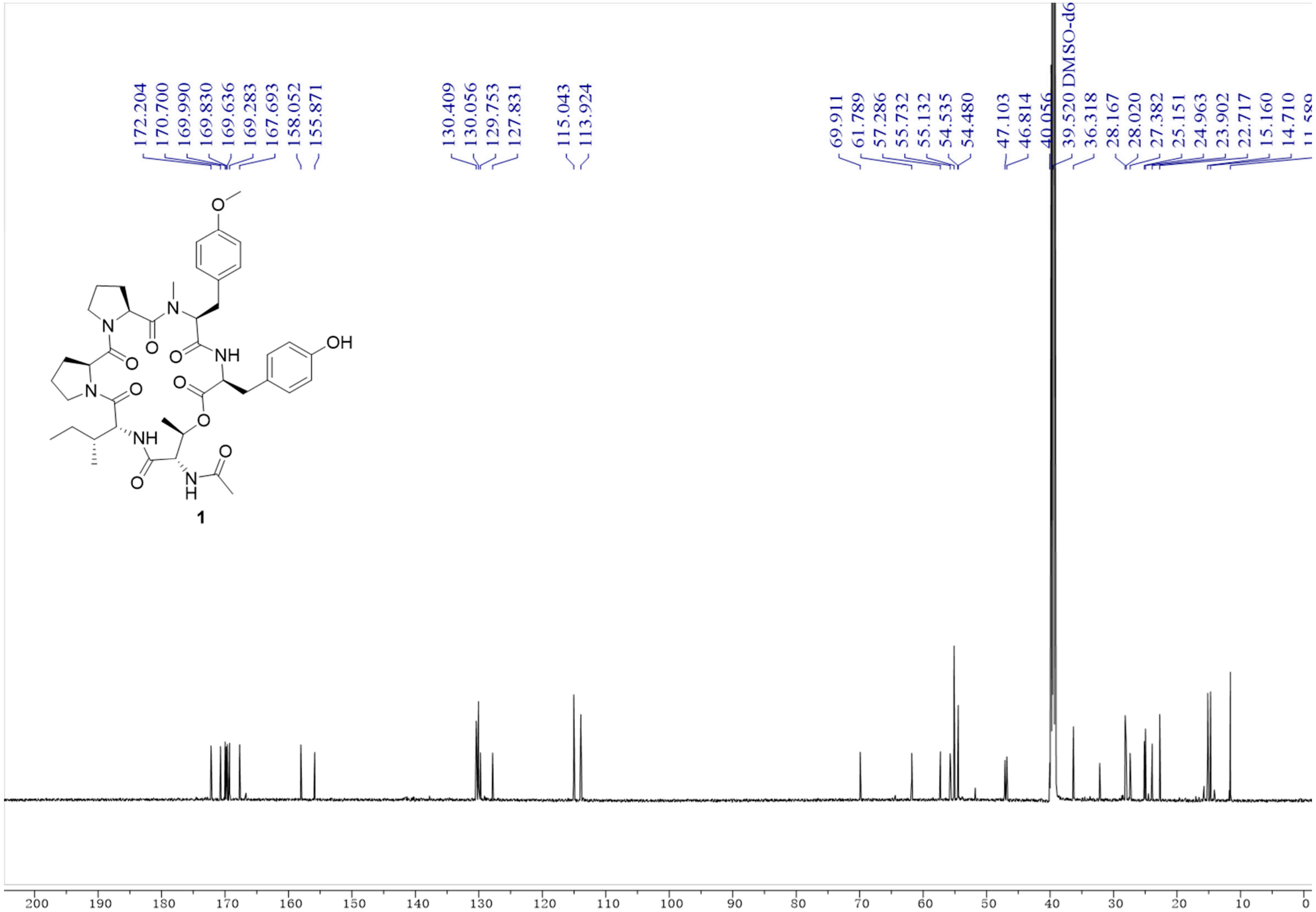

Figure S4 ^{13}C NMR spectrum of compound **1** in DMSO-d_6

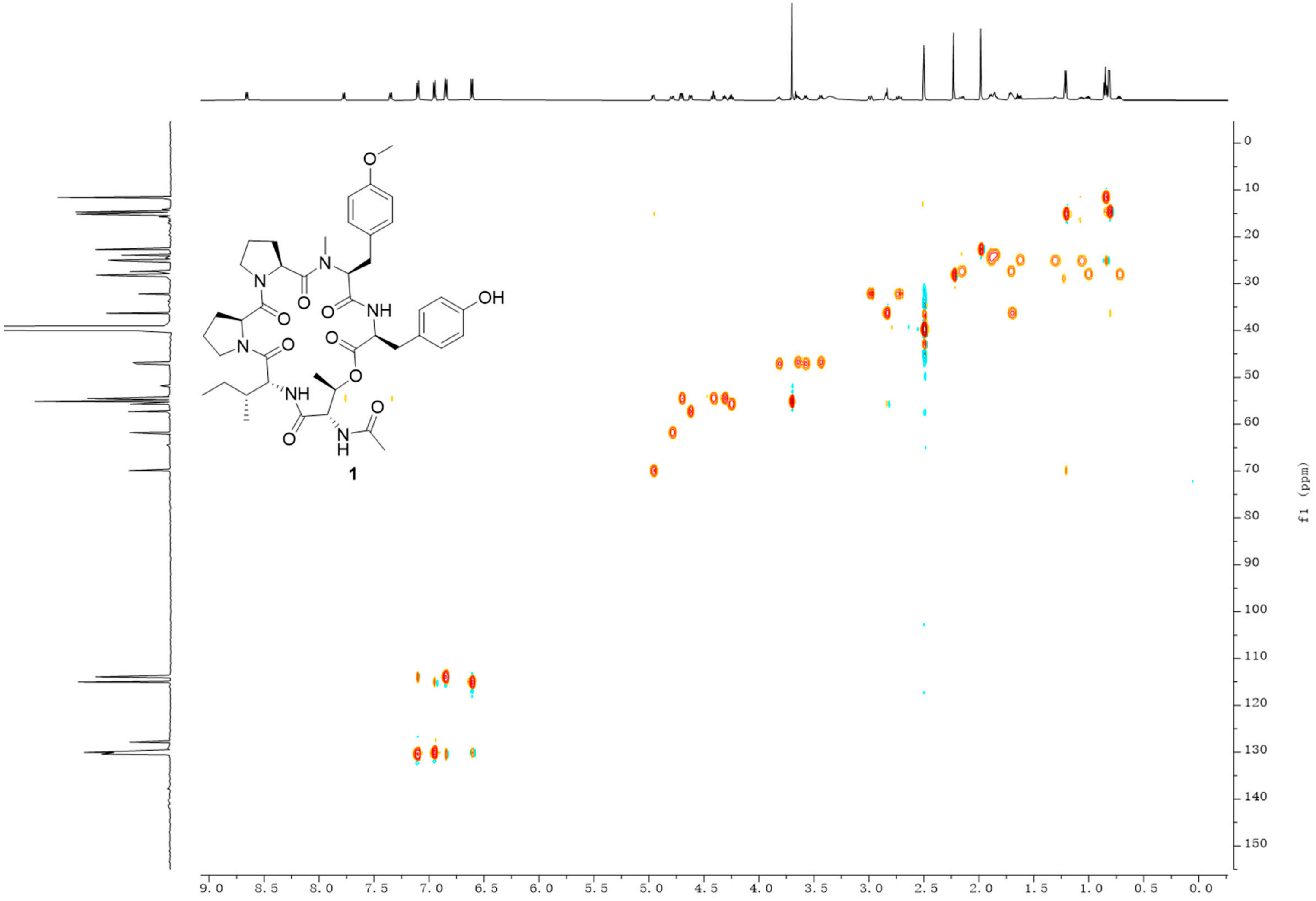

Figure S5 HSQC spectrum of compound **1** in DMSO-d_6

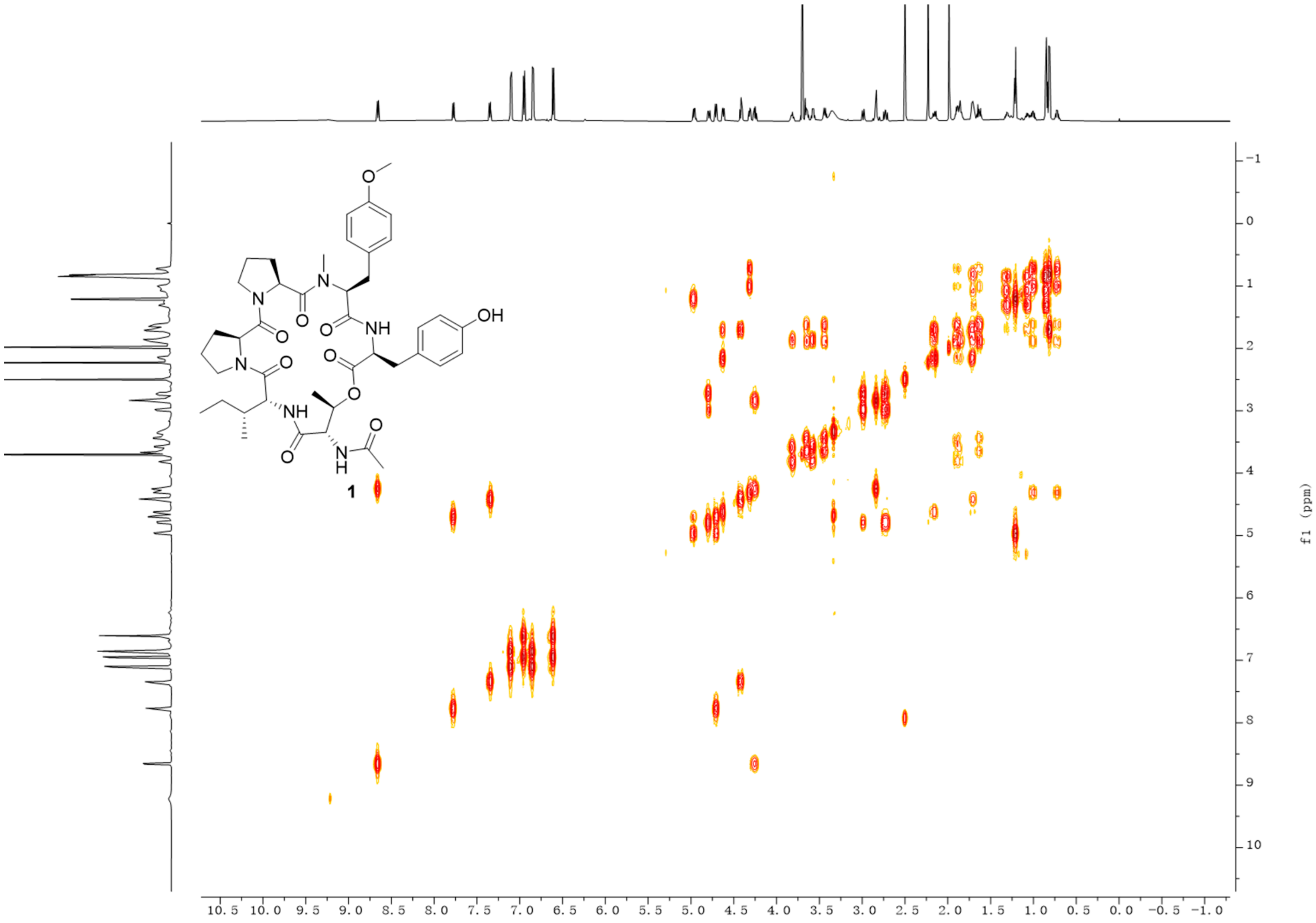

Figure S6 ^{1}H-^{1}H COSY spectrum of compound **1** in DMSO-*d*$_6$

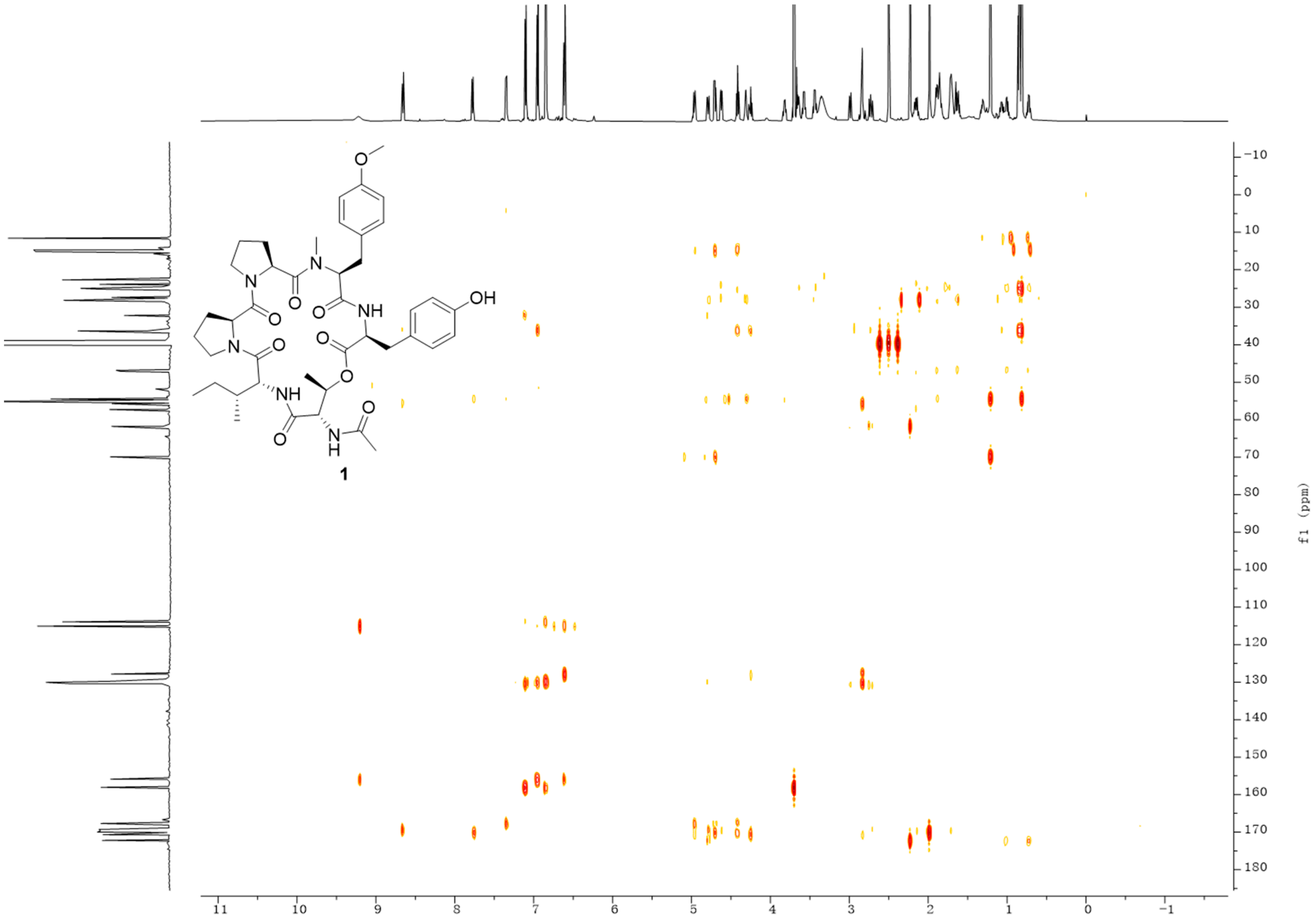

Figure S7 HMBC spectrum of compound **1** in DMSO-d_6

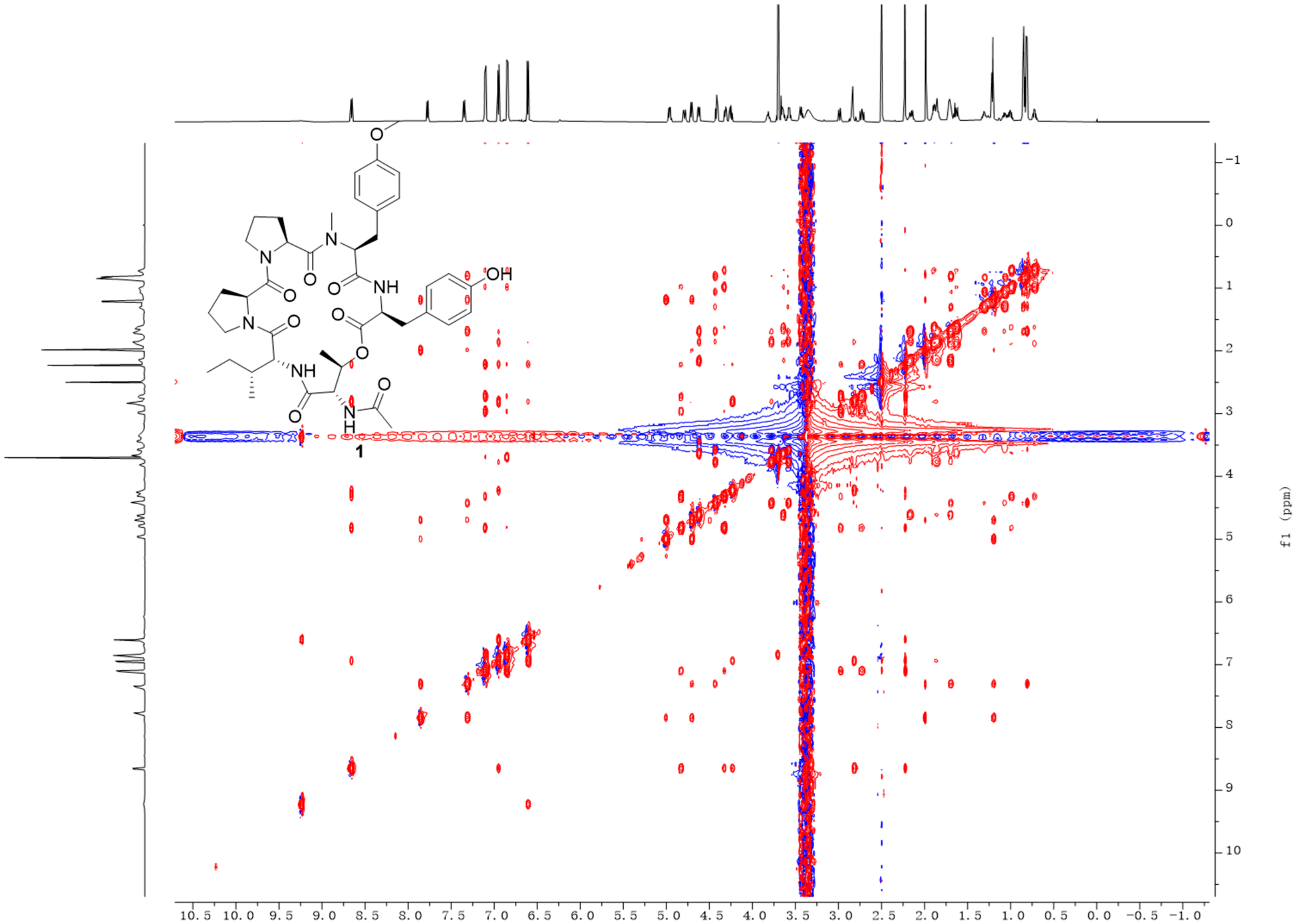

Figure S8 ROESY spectrum of compound **1** in DMSO-d_6

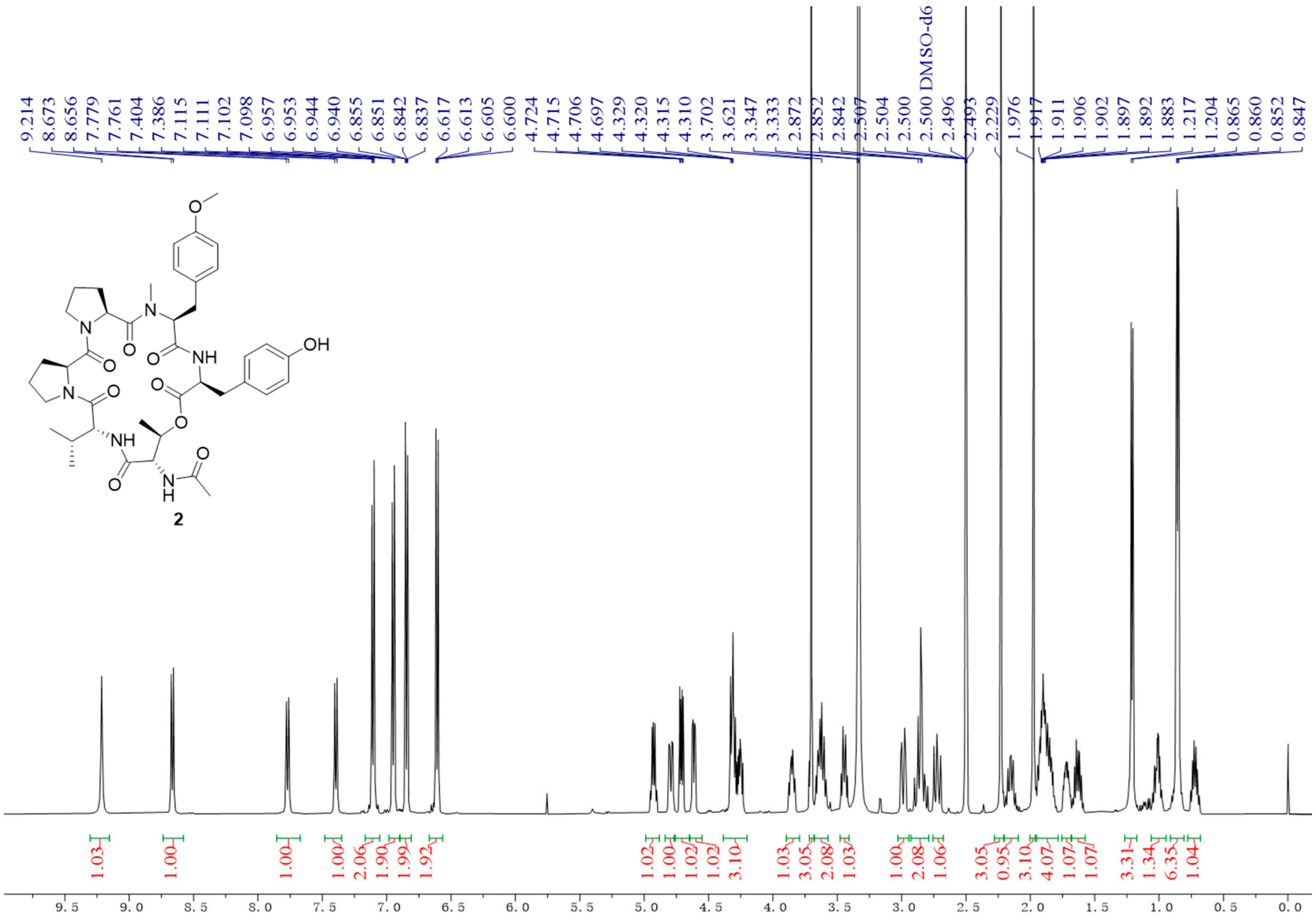

Figure S9 ^{1}H NMR spectrum of compound **2** in DMSO-d_6

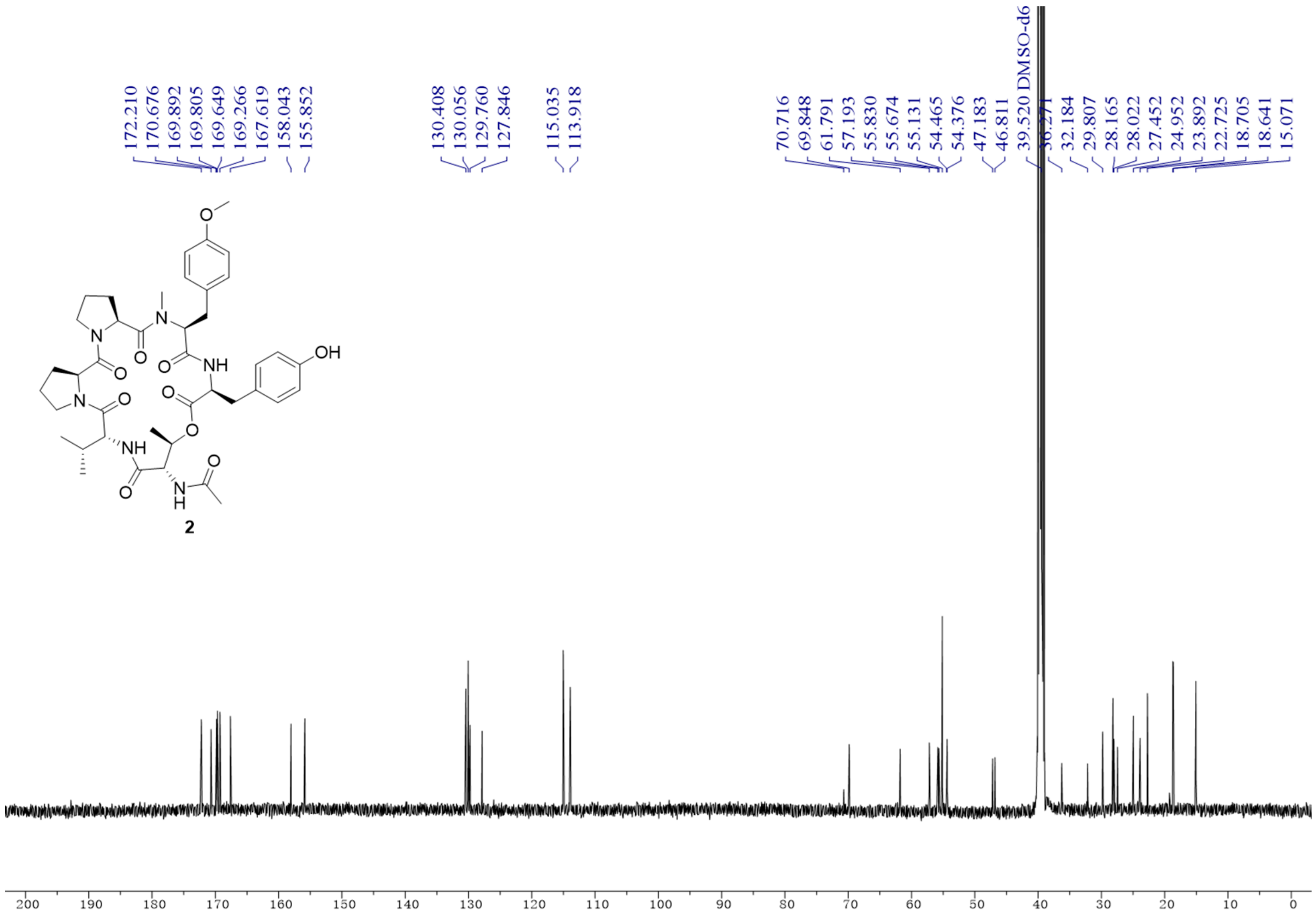

Figure S10 ^{13}C NMR spectrum of compound **2** in DMSO-d_6

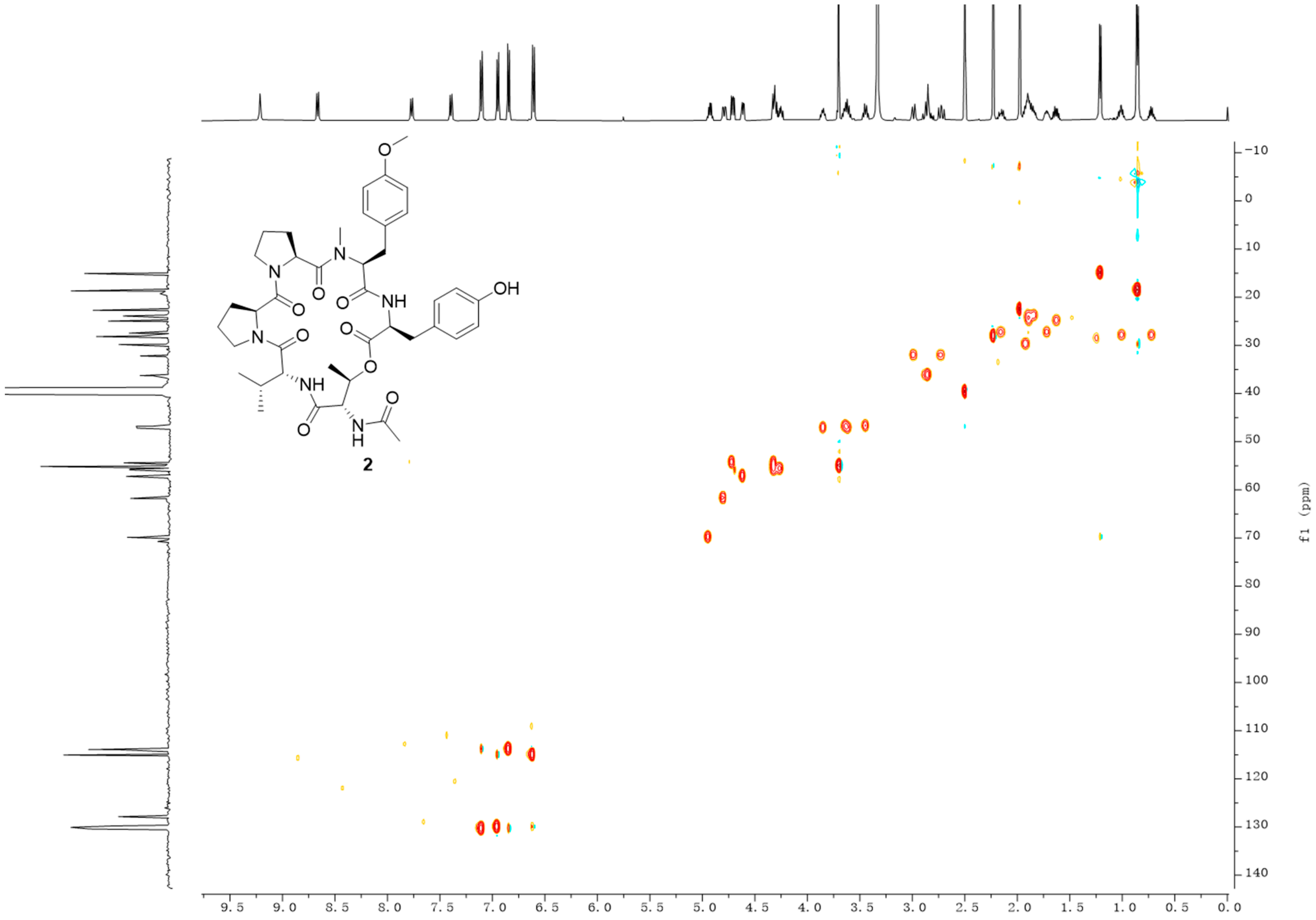

Figure S11 HSQC spectrum of compound **2** in DMSO-d_6

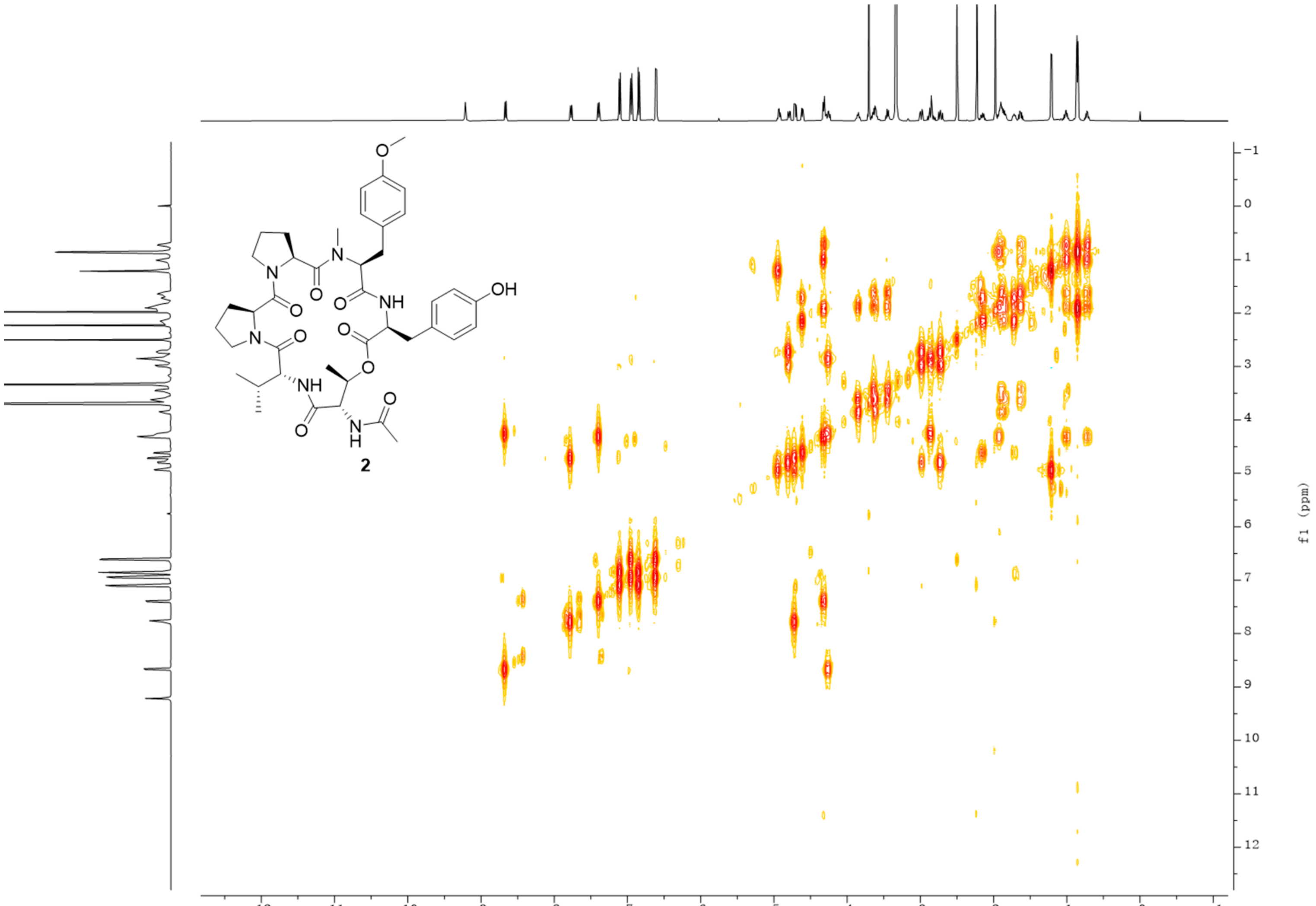

Figure S12 ^{1}H-^{1}H COSY spectrum of compound **2** in DMSO-d_6

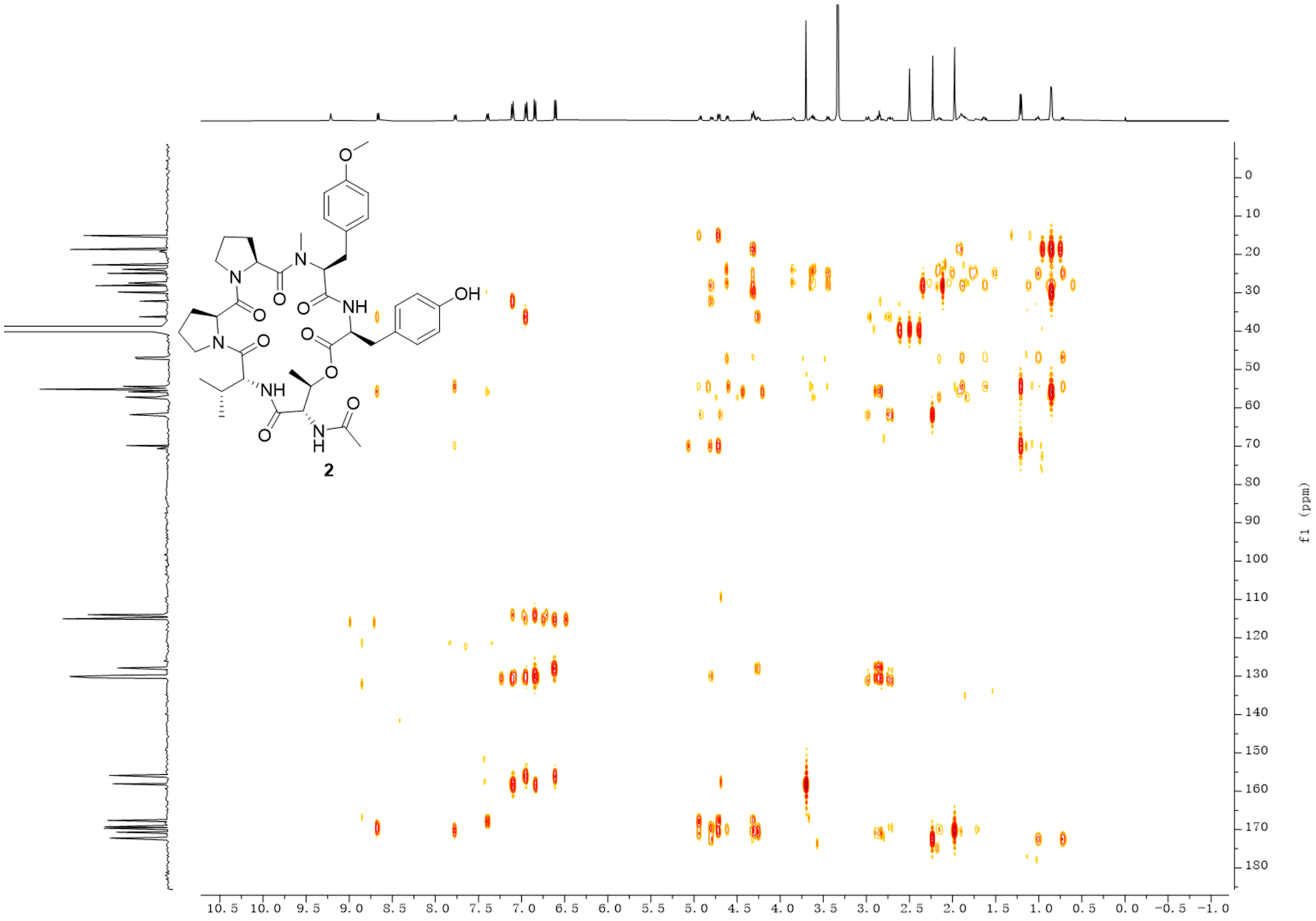

Figure S13 HMBC spectrum of compound **2** in DMSO-d_6

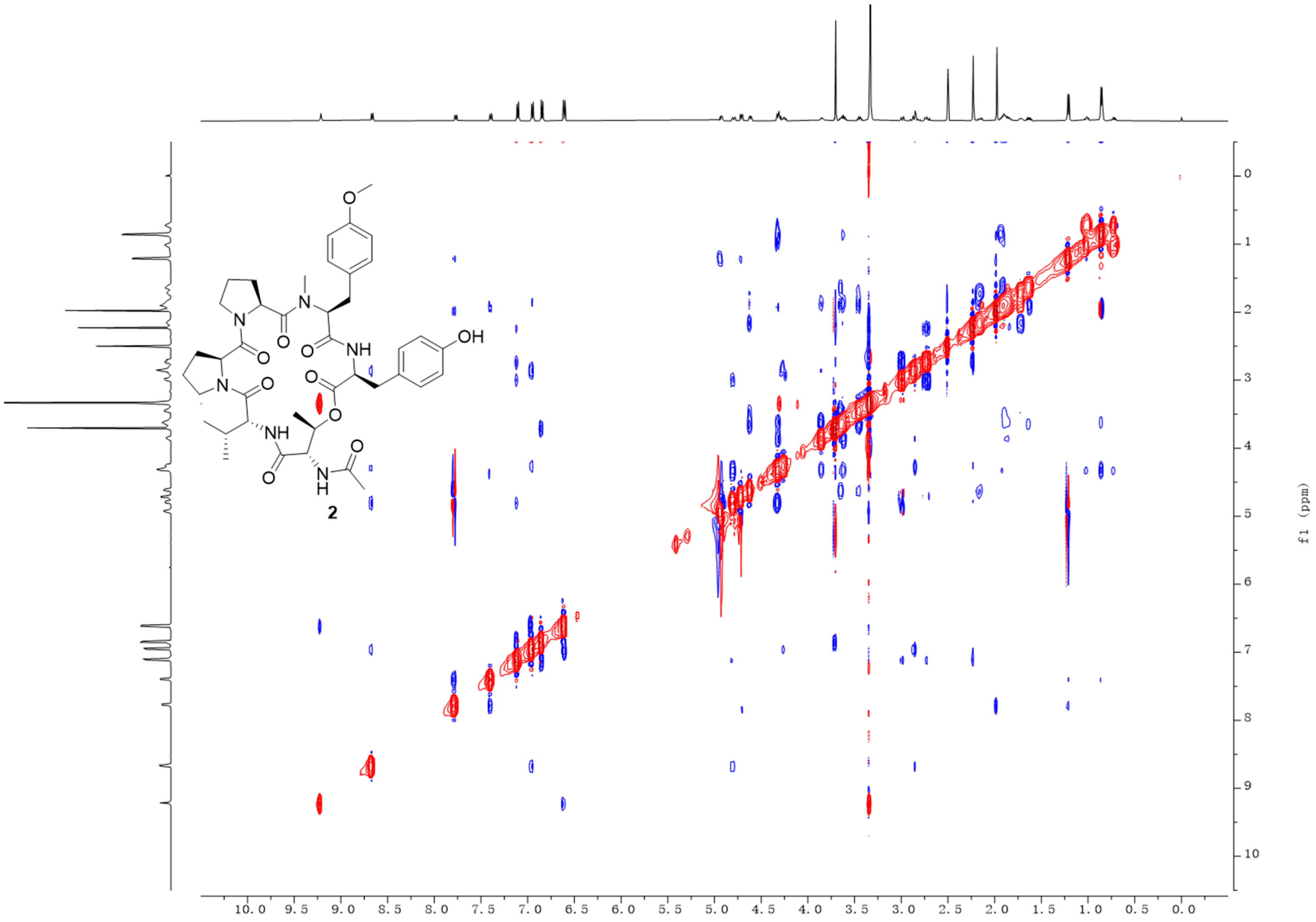

Figure S14 ROESY spectrum of compound **2** in DMSO-d_6
